# Factors that may affect the outcome of acute occlusive mesenteric ischemia. A single-center study

**DOI:** 10.1186/s12893-024-02310-9

**Published:** 2024-01-13

**Authors:** Qian Zhang, Tianyi Ma, Hongwei Zhao, Yuanxin Li, Peng Zhang

**Affiliations:** Department of Gastrointestinal Surgery, Beijing Tsinghua Changgung Hospital, School of Clinical Medicine, Tsinghua University, No. 168 Litang Road, Changping District, Beijing, 102218 China

**Keywords:** Factors, Acute mesenteric ischemia, Complication

## Abstract

**Background:**

Acute mesenteric ischemia is a rare but lethal disease. Acute occlusive mesenteric ischemia consists of mesenteric artery embolism, mesenteric artery thrombosis, and mesenteric vein thrombosis. This study aimed to investigate the factors that may affect the outcome of acute occlusive mesenteric ischemia.

**Methods:**

Data from acute occlusive mesenteric ischemia patients admitted between May 2016 and May 2022 were reviewed retrospectively. Patients were divided into 2 groups according to whether complications(Clavien‒Dindo ≥ 2) occurred within 6 months of the first admission. Demographics, symptoms, signs, laboratory results, computed tomography angiography features, management and outcomes were analyzed.

**Results:**

59 patients were enrolled in this study. Complications(Clavien‒Dindo ≥ 2) occurred within 6 months of the first admission in 17 patients. Transmural intestinal necrosis, peritonitis, white blood cell count, percentage of neutrophils, percentage of lymphocytes, neutrophil-to-lymphocyte ratio, lactate dehydrogenase, creatine kinase isoenzyme, cardiac troponin I, laparoscopic exploration rate, open embolectomy rate, enterostomy rate, length of necrotic small bowel, length of healthy small bowel, surgical time and intraoperative blood loss differed significantly between groups. Creatine kinase isoenzyme (OR = 1.415, 95% CI: 1.060–1.888) and surgical time (OR = 1.014, 95% CI: 1.001–1.026) were independent risk factors associated with complications(Clavien‒Dindo ≥ 2).

**Conclusions:**

Our analysis suggests that acute occlusive mesenteric ischemia patients with a creatine kinase isoenzyme level greater than 2.22 ng/mL or a surgical time longer than 156 min are more likely to experience complications’(Clavien‒Dindo ≥ 2) occurrence within 6 months of the first admission.

## Background

Acute mesenteric ischemia (AMI) is a rare disease. A British study [1] suggested that the incidence was 0.63/100,000/year. A study in Sweden [2] based on autopsy reports showed that the incidence was 12.90/100,000/year. Despite its low incidence, the mortality rate of this disease is as high as 50–69% [3]. AMI is categorized into four subtypes according to its cause: mesenteric artery embolism (EAMI), mesenteric artery thrombosis (TAMI), nonocclusive mesenteric ischemia (NOMI) and mesenteric vein thrombosis (VAMI). EAMI accounts for 25% of cases, TAMI accounts for approximately 40% of cases, VAMI accounts for approximately 15% of cases, and NOMI accounts for approximately 20% of cases [4]. Acute occlusive mesenteric ischemia (AOMI) consists of the EAMI, TAMI and VAMI. The incidence rate of complications ranges from 13.33–61.5% [5–10]. The complications include short bowel syndrome (SBS), electrolyte imbalance, intestinal obstruction, intestinal hemorrhage, renal or cardiac dysfunction, intestinal fistula and wound infection. Some of the complications are severe and may lead to death. According to the Clavien‒Dindo score system, patients with the score ≥ 2 have to be readmitted to the hospital, and the cost is high. Will different categories lead to different outcomes? Will early diagnosis and early management improve the outcomes? Will different operation methods decrease the complication rate? In our study, we aimed to identify significant factors that may affect the outcomes.

## Methods

### Case selection

Data from patients with AOMI admitted to the Beijing Tsinghua Changguang Hospital surgery emergency department or gastrointestinal surgery department from May 2016 to May 2022 were reviewed retrospectively. All diagnoses were confirmed by computed tomography angiography (CTA). The inclusion criteria were a diagnosis of superior mesenteric artery (SMA) embolism (CTA showed the emboli in SMA and the emboli located at least 3 cm far from the origin of SMA), SMA thrombosis (CTA showed the thrombus in SMA and the thrombus located within 3 cm far from the origin of SMA), or superior mesenteric vein (SMV) thrombosis (CTA showed the thrombus in SMV). The exclusion criteria were as follows: (1) age < 18 years; (2) patients who refused further treatment after diagnosis; or (3) diagnosis of NOMI (CTA showed bowel ischemia with no emboli or thrombus in SMA or SMV).

### Data collection

This study was approved by the Beijing Tsinghua Changgung Hospital Ethics Committee (22003-6-01). Informed consent was waived by the Beijing Tsinghua Changgung Hospital Ethics Committee. All methods were performed in accordance with the Declaration of Helsinki. All the cases were divided into 2 groups according to whether complications(Clavien‒Dindo ≥ 2) occurred within 6 months of the first admission. Cases without complications(Clavien‒Dindo ≥ 2) occurred within 6 months of the first admission were categorized into normal group. The other cases were categorized into complication group. Complications(Clavien‒Dindo ≥ 2) included SBS, electrolyte imbalance, intestinal obstruction, intestinal hemorrhage, renal or cardiac dysfunction, intestinal fistula and death. The following clinical characteristics were examined herein: age, sex, diagnosis, transmural intestinal necrosis (confirmed by the pathology reports), duration from onset to diagnosis, duration from onset to treatment, abdominal pain, abdominal distension, nausea and vomiting, hematemesis and hematochezia, diarrhea, comorbidities (cardiac problem including history of atrial fibrillation, recent myocardial infarction, cardiac thrombi, mitral valve disease, left ventricular aneurysm and endocarditis, previous embolic disease, diffuse atherosclerotic disease, portal hypertension, history of venous thromboembolism, oral contraceptives, estrogen use, thrombophilia pancreatitis), peritonitis, fever, white blood cell count (WBC), C reactive protein (CRP), hemoglobin (HGB), platelet (PLT), percentage of neutrophils (N%), percentage of lymphocytes (L%), neutrophil-to-lymphocyte ratio (NLR), D-dimer, lactate dehydrogenase (LDH), creatine kinase (CK), creatine kinase isoenzyme (CKMB), myoglobin (MYO), cardiac troponin I (CTNI), lactate (LAC), pondus hydrogenii (PH), CTA details (emboli or thrombus in vessel, decreased intestinal wall enhancement, intestinal wall thickening, pneumatosis intestinalis and ascites), surgical approach (endovascular surgery, laparoscopic exploration, open embolectomy and enterostomy), length of necrosis small bowel, length of healthy small bowel, surgical time and intraoperative blood loss.

### Treatment and follow-up

**Fig. 1:**
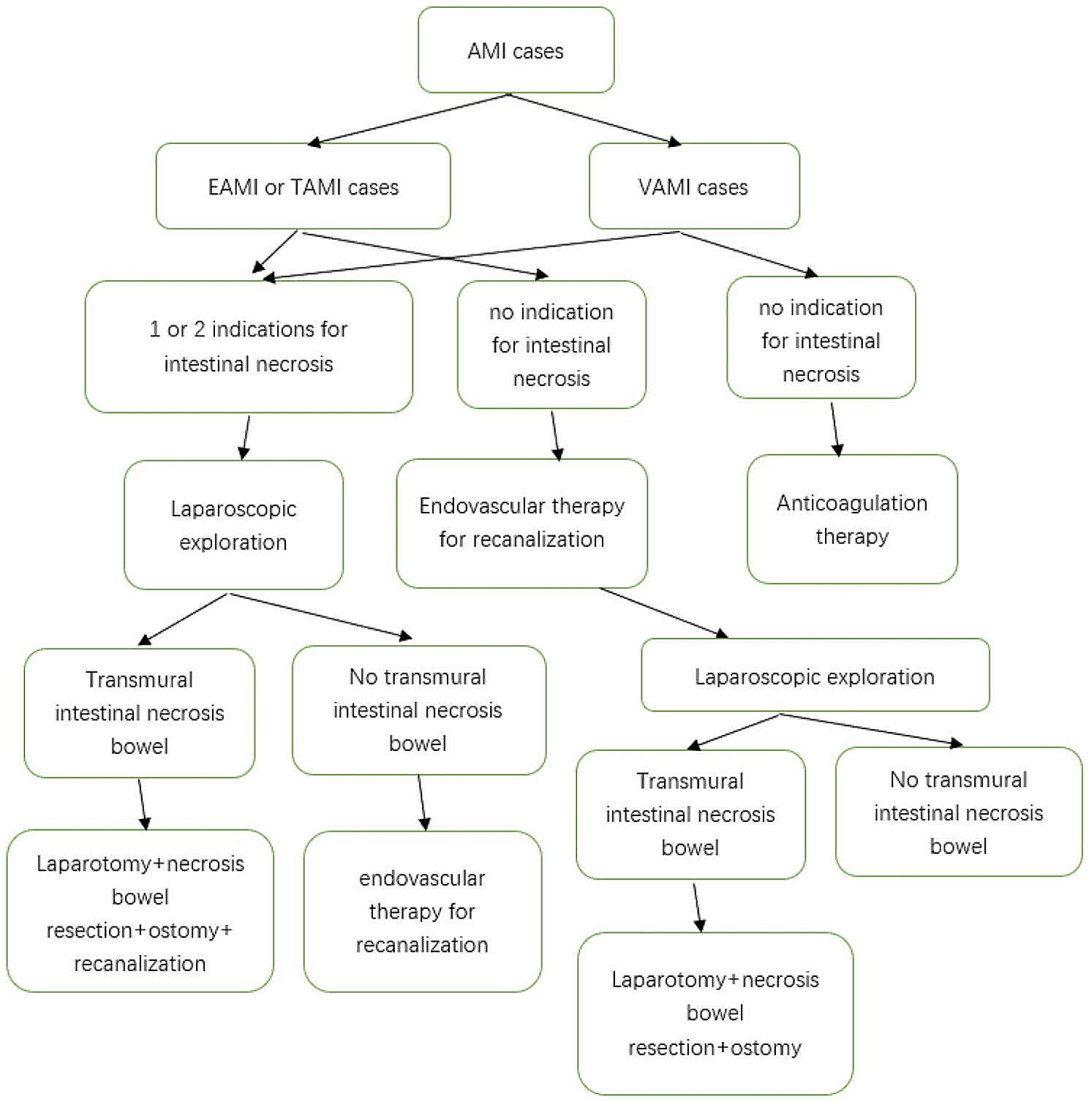
Management algorithm. AOMI cases treatment algorithm

We recommended AOMI cases followed our treatment algorithm as follows (Fig. 1).

The indications of intestinal necrosis with AOMI were as follows: (1) AOMI with decreased intestinal wall enhancement, intestinal wall thickening, pneumatosis intestinalis, or ascites on CTA; and (2) AOMI with peritonitis.

VAMI cases without indications of intestinal necrosis underwent anticoagulation therapy. EAMI or TAMI cases without indications of intestinal necrosis underwent endovascular procedures for recanalization. Subsequently, laparoscopic exploration was performed to confirm that there was no transmural intestinal necrosis.

AOMI patients with 1 or 2 indications of intestinal necrosis underwent laparoscopic exploration first. Intestinal necrosis was judged by inspection of the color of the intestines and intestinal peristalsis during the operation. Once transmural intestinal necrosis was confirmed, we converted to an open operation. Resection of the necrotic intestine and ostomy were performed. Otherwise, an endovascular procedure was performed for recanalization.

Anticoagulation, antibiotic therapy, rehydration, and nutrition were performed post-operation.

The criteria for discharge were as follows: (1) no remaining intestinal necrosis of the bowel; (2) total enteral nutrition tolerated; and (3) no need for intravenous antibiotics. All cases were followed up for 6 months. The primary endpoint was complications(Clavien‒Dindo ≥ 2) occurred within 6 months of the first admission.

### Statistical analysis

The factors were compared between groups. The results were analyzed by SPSS 25.0 (IBM, USA). A t test was used to compare normally distributed continuous variables, the Mann‒Whitney t test was used to compare non-normally distributed continuous variables. The chi-squared test was used to compare categorical data. Factors with significant difference were listed after t test and chi-squared test. Logistic regression (backward stepwise selection based on the likelihood ratio method) was performed to identify factors that were associated with complications among the listed factors. A receiver operating characteristic(ROC)curve was established for CKMB and surgical time. A statistically significant difference was indicated when *P* < 0.05.

## Results

**Fig. 2:**
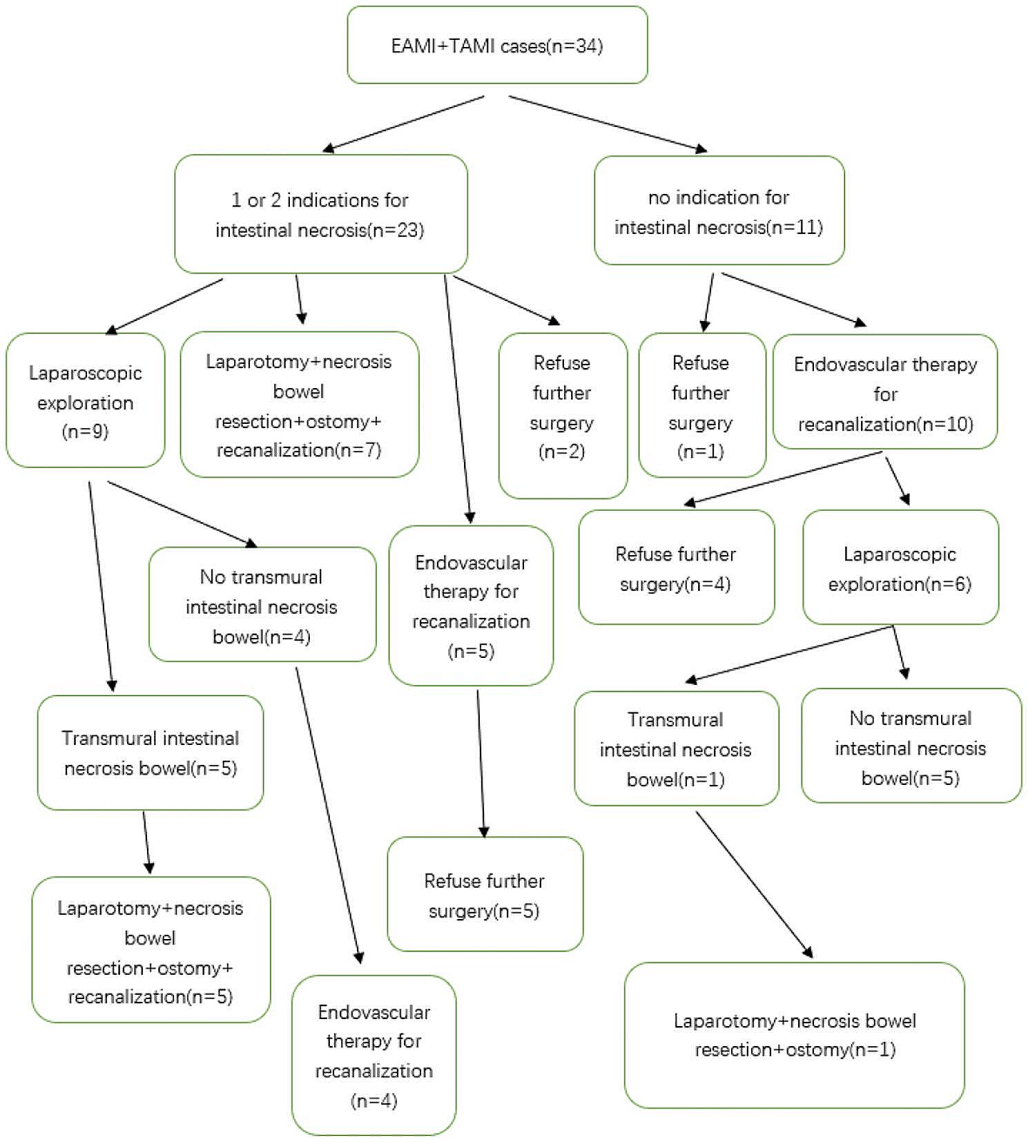
EAMI + TAMI cases management. Among the 34 patients with EAMI or TAMI, 11 cases had no indications for intestinal necrosis. 1 case refused further surgery and underwent anticoagulation therapy alone, the other 10 cases underwent endovascular surgery and all of them succeeded in recanalization. 4 of 10 cases refused further laparoscopic exploration, and the other 6 of 10 cases underwent laparoscopic exploration. Intestinal necrosis was found in 1 of 6 cases. Open surgery with necrotic small bowel removal and ostomy was performed. 23 cases with EAMI or TAMI had 1 or 2 indications for intestinal necrosis. 2 of 23 cases refused further surgery and underwent anticoagulation therapy alone. 5 of 23 cases underwent endovascular surgery for recanalization and refused further surgery after successful recanalization. 7 of 23 cases underwent open surgery and intestinal necrosis was found in all the cases. Open surgery with necrotic small bowel removal and ostomy was performed. 9 of 23 cases underwent laparoscopic exploration and intestinal necrosis was found in 5 of 9 cases. Open surgery with necrotic small bowel removal and ostomy was performed. The other 4 of 9 cases underwent endovascular surgery and all of them succeeded in recanalization

59 patients were enrolled in this study. 23 patients had EAMI, 11 patients had TAMI, and 25 patients had VAMI. The cases didn’t follow our treatment algorithm strictly. Among the 34 patients with EAMI or TAMI, 11 cases had no indications for intestinal necrosis. 1 case refused further surgery and underwent anticoagulation therapy alone, the other 10 cases underwent endovascular surgery and all of them succeeded in recanalization. 4 of 10 cases refused further laparoscopic exploration, and the other 6 of 10 cases underwent laparoscopic exploration. Intestinal necrosis was found in 1 of 6 cases. Open surgery with necrotic small bowel removal and ostomy was performed. 23 cases with EAMI or TAMI had 1 or 2 indications for intestinal necrosis. 2 of 23 cases refused further surgery and underwent anticoagulation therapy alone. 5 of 23 cases underwent endovascular surgery for recanalization and refused further surgery after successful recanalization. 7 of 23 cases underwent open surgery and intestinal necrosis was found in all the cases. Open surgery with necrotic small bowel removal and ostomy was performed. 9 of 23 cases underwent laparoscopic exploration and intestinal necrosis was found in 5 of 9 cases. Open surgery with necrotic small bowel removal and ostomy was performed. The other 4 of 9 cases underwent endovascular surgery and all of them succeeded in recanalization. The management was shown in Fig. 2.

**Fig. 3:**
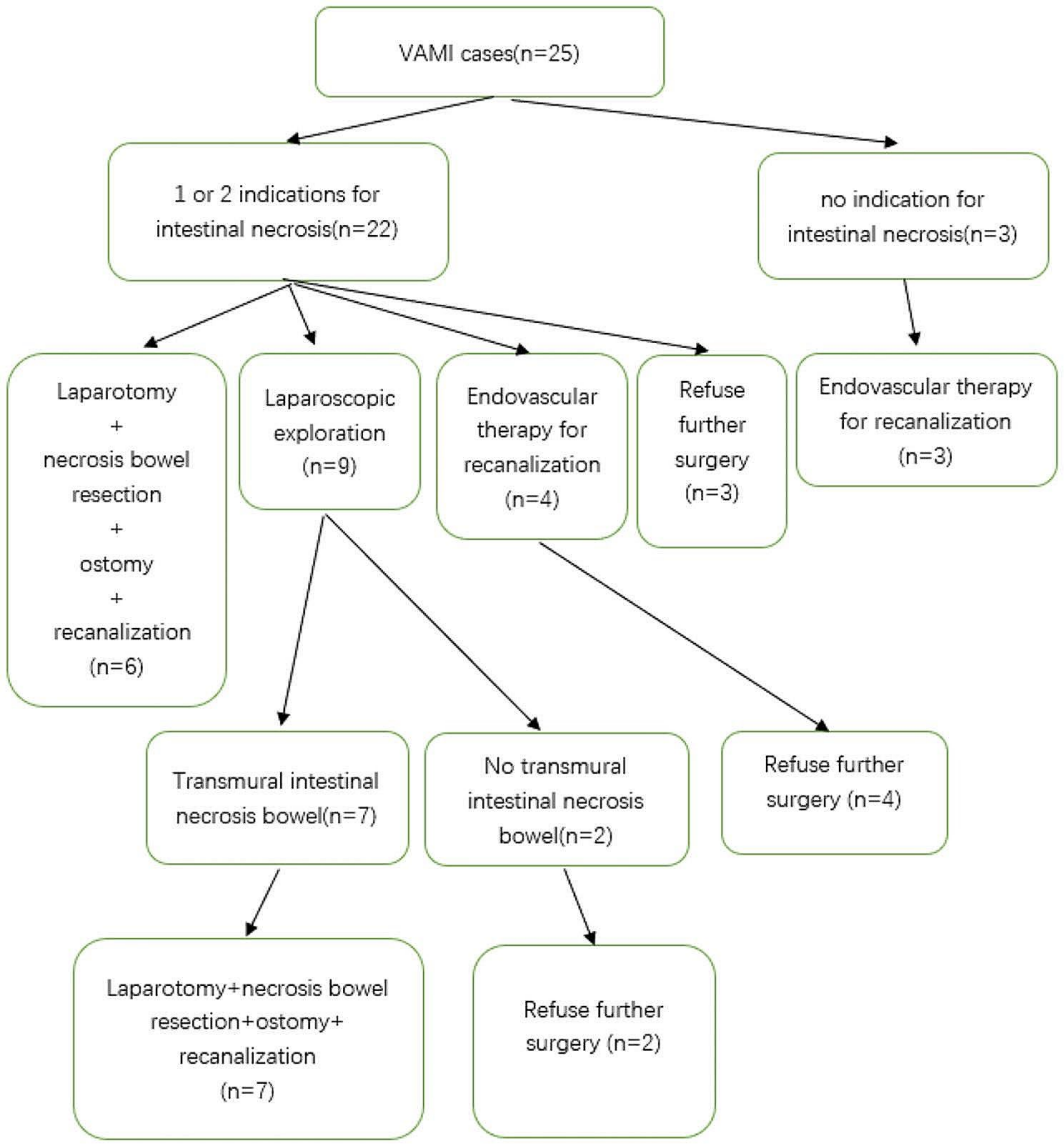
VAMI cases management. Among the 25 patients with VAMI, 3 cases had no indications for intestinal necrosis and underwent endovascular surgery. 22 cases had 1 or 2 indications for intestinal necrosis. 3 of 22 cases refused further surgery and underwent anticoagulation therapy alone. 4 of 22 cases underwent endovascular surgery for recanalization and refused further surgery after successful recanalization. 6 of 22 cases underwent open surgery and intestinal necrosis was found in all the cases. Open surgery with necrotic small bowel removal and ostomy was performed. 9 of 22 cases underwent laparoscopic exploration and intestinal necrosis was found in 7 of 9 cases. Open surgery with necrotic small bowel removal and ostomy was performed. The other 2 of 9 cases with no intestinal necrosis refused further surgery and underwent anticoagulation therapy

Among the 25 patients with VAMI, 3 cases had no indications for intestinal necrosis and underwent endovascular surgery. 22 cases had 1 or 2 indications for intestinal necrosis. 3 of 22 cases refused further surgery and underwent anticoagulation therapy alone. 4 of 22 cases underwent endovascular surgery for recanalization and refused further surgery after successful recanalization. 6 of 22 cases underwent open surgery and intestinal necrosis was found in all the cases. Open surgery with necrotic small bowel removal and ostomy was performed. 9 of 22 cases underwent laparoscopic exploration and intestinal necrosis was found in 7 of 9 cases. Open surgery with necrotic small bowel removal and ostomy was performed. The other 2 of 9 cases with no intestinal necrosis refused further surgery and underwent anticoagulation therapy. The management was shown in Fig. 3.

**Fig. 4:**
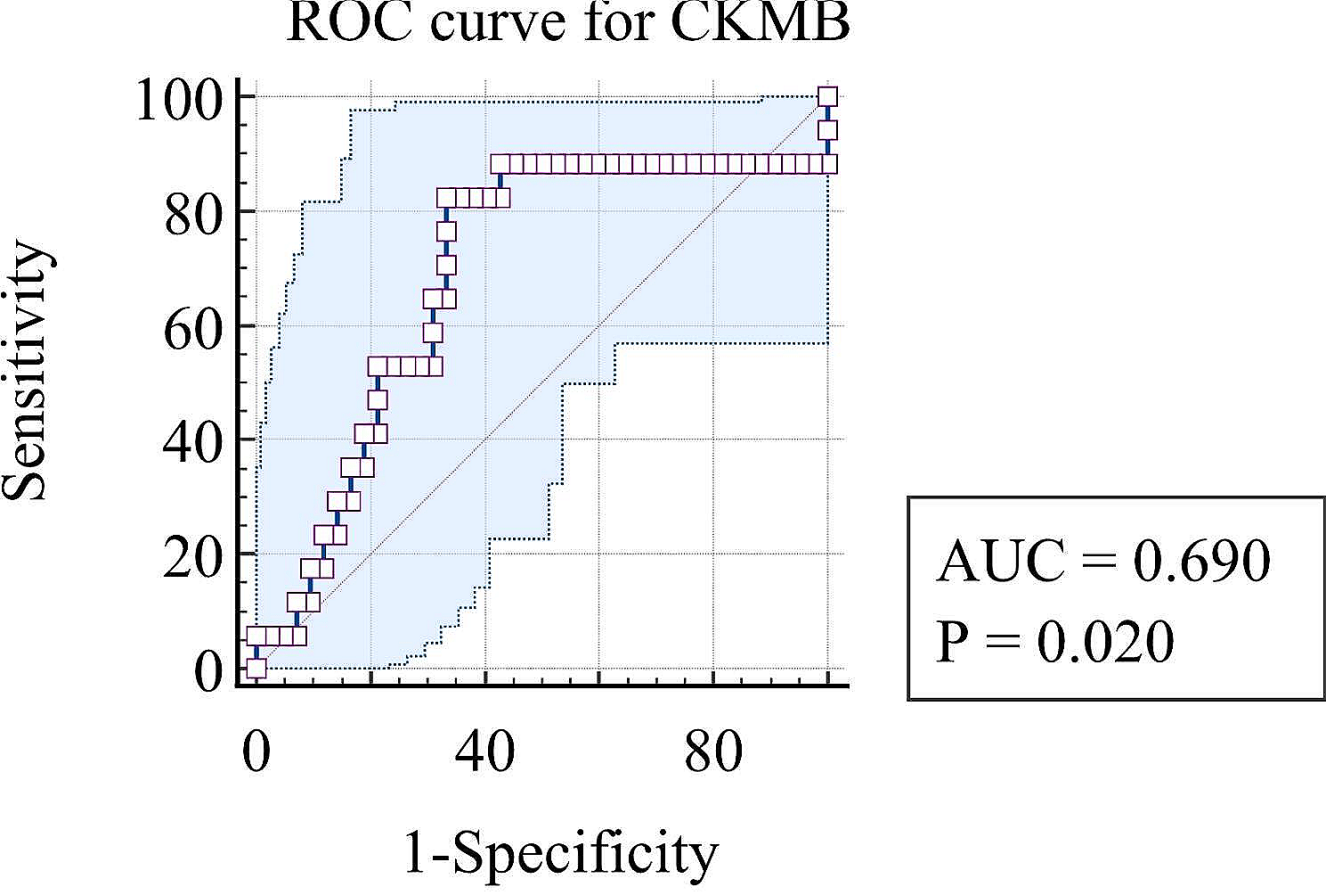
ROC curve for CKMB. ROC curve for CKMB suggested the area under the curve (AUC) values for CKMB was 0.69 (*P* = 0.02) and 0.814 (95% CI = 0.707–0.92)

**Fig. 5:**
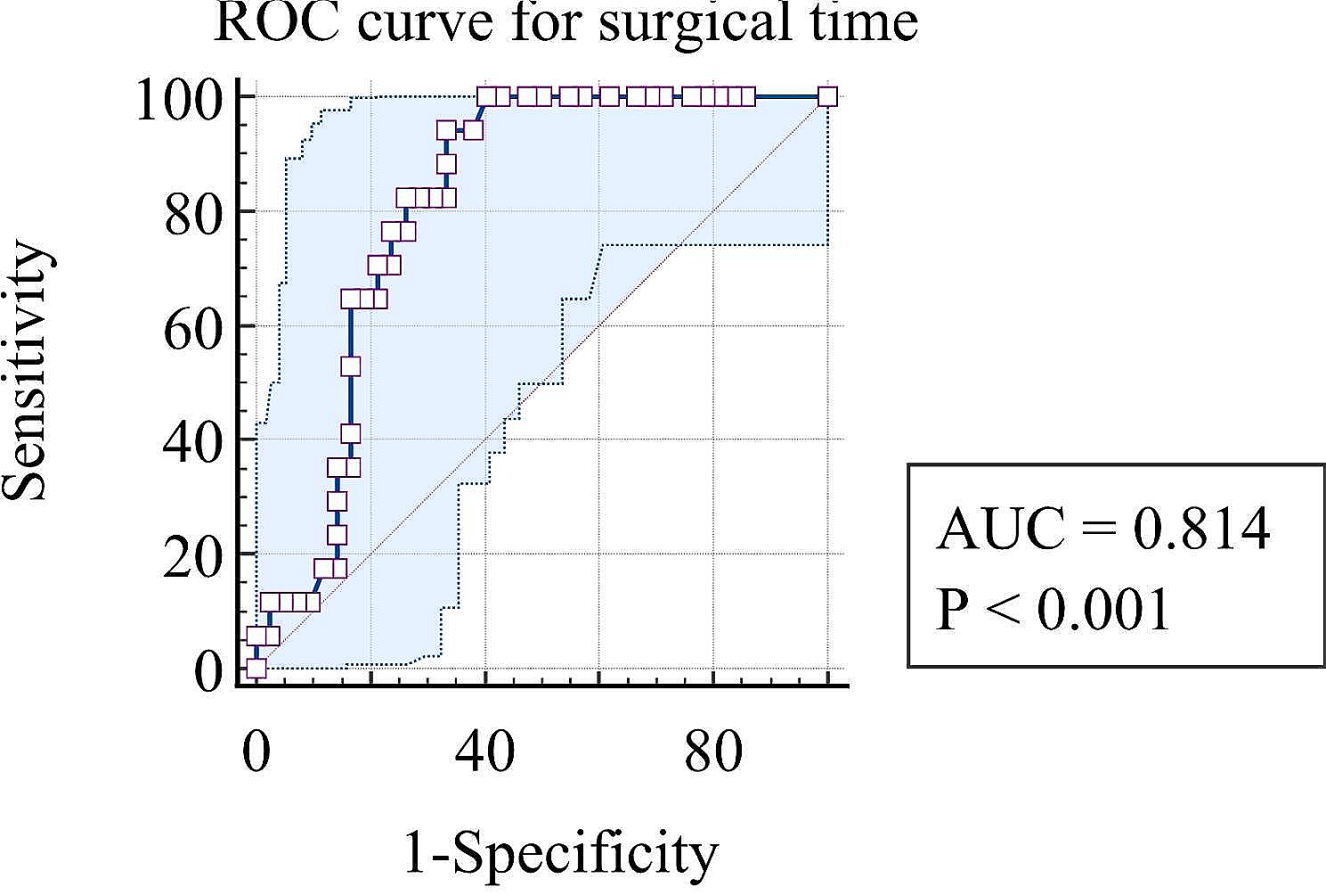
ROC curve for surgical time. ROC curve for surgical time suggested the area under the curve (AUC) values for surgical time was 0.814 (*P* < 0.001)

Severe complications within 6 months after the first admission occurred in 17 cases (12 males and 5 females) aged 67.94 ± 15.89 years. They were divided into the complication group. 2 patients died within 30 days post first management. 4 patients experienced short bowel syndrome and electrolyte imbalance. 2 patients experienced electrolyte imbalance, and 10 patients experienced intestinal obstruction. The other 42 patients were divided into the normal group. Compared to the normal group, the following parameters differed significantly after univariate analysis: the ratio of transmural intestinal necrosis (82.4% vs. 28.6%, *P* < 0.01), peritonitis (64.7% vs. 28.6%, *P* = 0.01), laparoscopic exploration (64.7% vs. 31%, *P* = 0.02), open embolectomy (82.4% vs. 26.2%, *P* < 0.01), and enterostomy (82.4% vs. 21.4%, *P* < 0.01). WBC (14.87 × 10^9^/L, 10.08 vs. 11.49 × 10^9^/L, 7.70, *P* = 0.03), N% (87.6%, 12.02 vs. 82.6%, 14.7, *P* = 0.03), L% (5.65%, 8.13 vs. 9.95%, 12.33, *P* = 0.03), NLR (16.2, 22.54 vs. 8.31, 13.42 *P* = 0.03), LDH (254.5 IU/L, 79.5 vs. 230.5 IU/L, 36.5, *P* = 0.03), CKMB (4.38 ng/ml, 5.39 vs. 1.71 ng/ml, 2.27, *P* = 0.02), CTNI (0.25 ng/ml, 0.09 vs. 0.14 ng/ml, 0.01, *P* = 0.02), length of necrosis small bowel (75 cm, 187.5 vs. 0 cm, 15, *P* < 0.01), length of healthy small bowel (325 cm, 221.5 vs. 400 cm, 0, *P* < 0.01), surgical time (300 min, 94.25 vs.105 min, 182.25, *P* < 0.01), and intraoperative blood loss (50 ml, 68.75 vs. 10 ml, 98, *P* = 0.01) also differed significantly. The results are shown in Table 1. Logistic regression (backward LR method) showed that CKMB (OR = 1.415, 95% CI = 1.060–1.888, *P* = 0.02) and surgical time (OR = 1.014, 95% CI = 1.001–1.026, *P* = 0.03) were independent risk factors associated with severe complications, as shown in Table 2. Additionally, most of the cases with elevated CKMB had cardiac problem.The ratio of it was much higher than that in cases with normal CKMB(82.4%vs 33.3%). Regarding the prediction of severe complications, ROC curves were drawn, and the area under the curve (AUC) values for CKMB and surgical time were 0.69 (95% CI = 0.533–0.848) and 0.814 (95% CI = 0.707–0.92), respectively, as shown in Figs. 4 and 5. When the cutoff for CKMB was 2.22 ng/ml, the sensitivity and specificity were 82.4% and 66.7%, respectively. When the cutoff for surgical time was 156 min, the sensitivity and specificity were 94.1% and 66.7%, respectively.

**Table 1:** Comparison of characteristics and outcomes between the complication groups

	Normal group *n* = 42	Complication group *n* = 17	P
Sex, male (*n*, %)	28, 66.70%	12, 70.60%	0.77
Age (year, mean ± SD)	62.64 ± 16.45	67.94 ± 15.89	0.26
Diagnosis			0.14
EAMI (*n*, %)	13, 31.00%	10, 58.80%	
TAMI (*n*, %)	9, 21.40%	2, 11.80%	
VAMI (*n*, %)	20, 47.60%	5, 29.40%	
Transmural intestinal necrosis (*n*, %)	12, 28.60%	14, 82.40%	< 0.01
Duration from onset to diagnosis (hours, Median, IQR)	84, 148.50	49, 90.00	0.74
Duration from onset to diagnosis categories			0.98
≤ 24 h (*n*, %)	15, 35.70%	6, 35.30%	
> 24 h (*n*, %)	27, 64.30%	11, 64.70%	
Duration from onset to treatment (hours, Median, IQR)	98.75, 207.13	75.75, 140.13	0.91
Duration from onset to treatment categories			0.38
≤ 24 h(*n*, %)	8, 19.00%	1, 5.90%	
> 24 h (*n*, %)	34, 81.00%	16, 94.10%	
Abdominal pain (*n*, %)	41, 97.60%	17, 100.00%	1.00
Abdominal distension (*n*, %)	28, 66.70%	15, 88.20%	0.17
Nausea (*n*, %)	24, 57.10%	13, 76.50%	0.16
Vomiting (*n*, %)	19, 45.20%	12, 70.60%	0.08
Hematemesis (*n*, %)	3, 7.10%	3, 17.60%	0.46
Hematochezia (*n*, %)	14, 33.30%	7, 41.20%	0.57
Diarrhea (*n*, %)	5, 11.90%	2, 11.80%	1.00
Comorbidities (*n*, %)	35, 83.30%	16, 94.10%	0.50
Peritonitis (*n*, %)	12, 28.60%	11, 64.70%	0.01
Fever (*n*, %)	12, 28.60%	7, 41.20%	0.35
WBC (10^9^/L, Median, IQR)	11.49, 7.70	14.87, 10.08	0.03
CRP (mg/L, Median, IQR)	63, 113.37	47.18, 102.07	0.87
PLT(10^9^/L, Median, IQR)	210.50, 69.75	202, 83.75	0.51
HGB (mg/L, mean ± SD)	130.98 ± 27.26	141.56 ± 24.10	0.13
N% (%,Median, IQR)	82.60, 14.70	87.60, 12.02	0.03
L% (%,Median, IQR)	9.95, 12.33	5.65, 8.13	0.03
NLR (Median, IQR)	8.31, 13.42	16.20, 22.54	0.03
D-dimer (mg/L, Median, IQR)	6.72, 11.58	5.73, 9.36	0.85
LDH(IU/L, Median, IQR)	230.50, 36.50	254.50, 79.50	0.03
CK(IU/L, Median, IQR)	100.50, 112.50	194, 248.75	0.26
CKMB(ng/mL, Median, IQR)	1.71, 2.27	4.38, 5.39	0.02
MYO(ng/mL, Median, IQR)	59.78,106.20	112.50, 125.84	0.12
CTNI(ng/mL, Median, IQR)	0.14, 0.01	0.25, 0.09	0.02
LAC(mmol/L, Median, IQR)	1.85, 0.90	2.20, 1.63	0.22
PH(Median, IQR)	7.40, 0.06	7.42, 0.06	0.70
CTA			
Emboli or thrombus in vessel (*n*, %)	42,100.00%	17,100.00%	
Decreased intestinal wall enhancement (*n*, %)	14, 33.30%	9, 52.90%	0.16
Intestinal wall thickening (*n*, %)	28, 66.70%	13, 76.50%	0.46
Pneumatosis intestinalis (*n*, %)	0, 0.00%	1, 5.90%	0.29
Ascites (*n*, %)	16, 38.10%	9, 52.90%	0.30
Surgical approach (*n*, %)	35, 83.30%	17,100.00%	0.18
Endovascular surgery (*n*, %)	21, 50.00%	6, 35.30%	0.30
Laparoscopic exploration (*n*, %)	13, 31.00%	11, 64.70%	0.02
Open embolectomy (*n*, %)	11, 26.20%	14, 82.40%	<0.01
Enterostomy (*n*, %)	9, 21.40%	14, 82.40%	<0.01
Length of necrosis small bowel(cm, Median, IQR)	0, 15.00	75, 187.50	<0.01
Length of healthy small bowel(cm, Median, IQR)	400, 0.00	325, 221.50	<0.01
Surgical time(min, Median, IQR)	105, 182.25	300, 94.25	<0.01
Intraoperative blood loss(ml, Median, IQR)	10, 98.00	50, 68.75	0.01

**Table 2:** Factors associated with complications after logistic regression

Factor	Wald	P	OR	95% CI
Transmural intestinal necrosis	0.000	1.00		
Laparoscopic exploration	2.596	0.11		
Enterostomy	0.000	1.00		
CKMB	5.563	0.02	1.415	1.060–1.888
Length of necrosis small bowel	1.490	0.22		
Surgical time	4.756	0.03	1.014	1.001–1.026

## Discussion

AMI is a rare but lethal disease. The reported mortality within 30 postoperative days ranged from 8.9 to 73.5% during the past decade [5, 8–22]. The incidence rate of complications ranged from 13.33–61.5% [5–10]. The complications include SBS, electrolyte imbalance, intestinal obstruction, intestinal hemorrhage, renal or cardiac dysfunction, intestinal fistula and wound infection. Some of the complications lead to readmission and high costs. Previous studies have investigated the predictive factors of transmural intestinal necrosis in AMI. Previous studies have also investigated the predictive factors of in-hospital mortality. However, few studies have focused on prognostic factors for outcome. This study aimed to identify factors that may be associated with complications (Clavien‒Dindo ≥ 2) that require readmission.

When transmural intestinal necrosis occurs, exudation around the bowel presents, and peritonitis appears. The WSES guidelines suggested that prompt laparotomy should be performed for AMI patients with overt peritonitis due to the high possibility of bowel necrosis [23]. This study demonstrated that more peritonitis occurred in the complication group. However, multivariate analysis showed that peritonitis was not an independent risk factor associated with complications. Transmural intestinal necrosis leads to bowel resection. A large amount of small bowel resection leads to inadequate absorption and electrolyte imbalance, even SBS. A previous study reported that the incidence of SBS caused by AMI was 25–30% [24]. In our study, cases in the complication group had a higher ratio of transmural intestinal necrosis, and the necrotic bowel was longer. However, none of them were predictive factors of severe complications. This was mainly because of the 3 points. The healthy bowel left was more than 100 cm, the colon was not involved, and there was late reconstruction of the bowel continuity.

Delayed diagnosis and management are associated with intestinal necrosis and in-hospital mortality [25]. Mikail Cakir et al. reported that irreversible intestinal mucosal necrosis occurred 4 h after occlusion of the superior mesenteric artery in a rat model [26]. The literature reported that the time from diagnosis to management ranged from 27 to 120 h [6, 13, 27]. Mateusz Jagielski et al. reported that the mortality rate was 100% if the time from diagnosis to management exceeded 24 h [8]. In our study, there were no significant differences in the duration from symptom onset to diagnosis or the duration from onset to treatment. It might because that the criteria for grouping were not only 30-day post management mortality but also other complications (Clavien‒Dindo ≥ 2). Most of the cases in this study took more than 24 h from diagnosis to management also might influence the result in our study.

When the patients admitted to the ER department with suspected AMI, most doctors routinely ordered complete blood cell count, biochemistry, D-dimer, and arterial blood gas analysis. The sensitivity and specificity of these biomarkers remain debated. Some studies reported that WBC, CRP, NLR, red blood cell volume distribution width (RDW), total bilirubin, creatinine, lactate, pH and PLT were significantly different between the intestinal necrosis group and the short-term postoperative death group [12, 22]. Other studies reported that a low pH level, low lymphocyte count, low platelet count, high platelet volume distribution width (PDW) level, high platelet-to-lymphocyte ratio and high creatinine level were risk factors associated with intestinal necrosis and short-term postoperative death [11, 15, 17, 18, 20, 28, 29]. In contrast, another study reported that routinely used laboratory tests could not predict intestinal necrosis or postoperative death [7, 8, 14, 30]. This study suggested that WBC, N%, L%, NLR, LDH, CKMB, and CTNI differed significantly in the complication group. Few studies have reported that CKMB and CTNI could predict mortality or poor outcomes. Both of them were used to suggest cardiac injury. Most of the cases with elevated CKMB in our study had cardiac problem.The ratio of it was much higher than that in cases with normal CKMB(82.4%vs 33.3%). It would reduce the patient’s tolerance to infection, surgery, and ischemia, and increase the difficulty of postoperative recovery. Our results demonstrated that AOMI with cardiac injury might lead to poor outcome. Logistic regression showed that CKMB was an independent risk factor associated with complications(Clavien‒Dindo ≥ 2) in this study. When the cutoff for CKMB was 2.22 ng/ml, the sensitivity and specificity were 82.4% and 66.7%, respectively.

CTA have become the standard to identify AMI recently. Prasaanthan Gopee-Ramanan et al. reported that the accuracy of CTA for identifying AMI was 92.9% [31]. Decreased intestinal wall enhancement, mesenteric stranding, dilated bowel and ascites pneumatosis intestinalis were reported in CTA with intestinal necrosis [13, 15, 16, 32]. Mothes, H. et al. reported that the specificities of decreased intestinal wall enhancement, pneumatosis intestinalis, and mesenteric stranding in predicting intestinal necrosis were 88.6%, 98.6% and 77.1%, respectively [13]. Wang, X. et al. reported that pneumatosis intestinalis (OR = 7.08) and ascites (OR = 9.49) were independent risk factors for intestinal necrosis [15]. In our study, none of the CTA findings differed between groups; therefore, proper management could decrease the complication rate even with bowel necrosis.

The surgical approach for AMI needs to achieve 3 goals: revascularization, resection of necrotic bowel and restoration of viable bowel as long as possible. Compared with endovascular surgery, open surgery leads to a higher rate of complications [33]. We selected enterostomy after necrotic bowel removal because of the fear of intestinal fistula after one-stage anastomosis. It was reported that the rate ranged from 23.4–27% [34, 35]. Enterostomy leads to electrolyte imbalance due to the loss of a large amount of digestive juice, especially in patients with improper home enteral nutrition. That was why our study revealed a higher ratio of enterostomy in the complication group. Although the heathy bowel length was shorter in the complication group, it failed to be a predictive factor associated with complications. This was because the length of the healthy bowel was more than 100 cm and no colon was involved. Unlike other reports, our study revealed that surgical time was an independent risk factor for complications(Clavien‒Dindo ≥ 2). Prolonged surgical time usually caused by open surgery which led to a higher rate of complications [33] in our study. Prolonged surgical time led to prolonged intestinal ischemia time and prolonged severe infection time, and finally led to poor outcome. When the cutoff for surgical time was 156 min, the sensitivity and specificity were 94.1% and 66.7%, respectively.

### Limitations

Since this study was a retrospective study with a small sample size, the results need to be tested with studies including a larger number of cases.

## Conclusions

In our study, AOMI patients with a CKMB level of more than 2.22 ng/mL or a surgical time of more than 156 min are more likely to experience complications’(Clavien‒Dindo ≥ 2) occurrence within 6 months of the first admission.

## Data Availability

The data used and/or analyzed during the current study are available from the corresponding author upon reasonable request.

## Abbreviations

AMI: Acute mesenteric ischemia
AOMI: Acute occlusive mesenteric ischemia
EAMI: Mesenteric artery embolism
TAMI: Mesenteric artery thrombosis
VAMI: Mesenteric vein thrombosis
NOMI: Nonocclusive mesenteric ischemia
CTA: Computed tomography angiography
WBC: White blood cell count
N%: Percentage of neutrophils
L%: Percentage of lymphocytes
NLR: Neutrophil-to-lymphocyte ratio
LDH: Lactate dehydrogenase
CKMB: Creatine kinase isoenzyme
CTNI: Cardiac troponin I
SBS: Short bowel syndrome
CRP: C reactive protein
HGB: Hemoglobin
PLT: Platelet
CK: Creatine kinase
MYO: Myoglobin
LAC: Lactate
PH: Pondus hydrogenii
SMA: Superior mesenteric artery
SMV: Superior mesenteric vein
RDW: Red blood cell volume distribution width
PDW: Platelet volume distribution width
